# Trends in Antiretroviral Therapy Eligibility and Coverage Among Children Aged <15 Years with HIV Infection — 20 PEPFAR-Supported Sub-Saharan African Countries, 2012–2016

**DOI:** 10.15585/mmwr.mm6719a4

**Published:** 2018-05-18

**Authors:** Amanda Burrage, Monita Patel, Kelsey Mirkovic, Eric Dziuban, Wondimu Teferi, Laura Broyles, Emilia Rivadeneira

**Affiliations:** ^1^Epidemic Intelligence Service, CDC; ^2^Division of Global HIV and Tuberculosis, CDC; ^3^Division of Global HIV and Tuberculosis, CDC Botswana; ^4^Division of Global HIV and Tuberculosis, CDC Ethiopia.

Rapid disease progression and associated opportunistic infections contribute to high mortality rates among children aged <15 years with human immunodeficiency virus (HIV) infection (*1*). Antiretroviral therapy (ART) has decreased childhood HIV-associated morbidity and mortality rates over the past decade (*2*). As accumulating evidence revealed lower HIV-associated mortality with early ART initiation, the World Health Organization (WHO) guidelines broadened ART eligibility for children with HIV infection (*2*). Age at ART initiation for children with HIV infection expanded sequentially in the 2010, 2013, and 2016 WHO guidelines to include children aged <2, <5, and <15 years, respectively, regardless of clinical or immunologic status (*3*–*5*). The United States President’s Emergency Plan for AIDS Relief (PEPFAR) has supported ART for children with HIV infection since 2003 and, informed by the WHO guidelines and a growing evidence base, PEPFAR-supported countries have adjusted their national pediatric guidelines. To understand the lag between guideline development and implementation, as well as the ART coverage gap, CDC assessed national pediatric HIV guidelines and analyzed Joint United Nations Programme on HIV and AIDS (acquired immunodeficiency syndrome; UNAIDS) data on children aged <15 years with HIV infection and the numbers of these children on ART. Timeliness of WHO pediatric ART guideline adoption varied by country; >50% of children with HIV infection are not receiving ART, underscoring the importance of strengthening case finding and linkage to HIV treatment in pediatric ART programs.

Pediatric ART eligibility criteria during 2012–2016 were abstracted from published national HIV treatment guidelines of 20 PEPFAR-supported countries in sub-Saharan Africa with the highest pediatric HIV burden.* Pediatric ART eligibility was defined as the recommended age for ART initiation, regardless of clinical or immunologic status, and was categorized by the following age categories: <1, <2, <5, or <15 years. Countries with “treat all” ART eligibility (i.e., immediate ART eligibility for all persons with HIV infection regardless of age, or clinical or immunologic status) for all persons living with HIV were categorized as <15 years, because ages ≥15 were not included in this analysis. The year of the ART eligibility policy or the date of another published document that specified a change in pediatric ART eligibility guidelines was considered the national HIV guideline publication date.

UNAIDS data for the 20 countries were abstracted from the publicly available AIDSinfo website following the release of 2016 UNAIDS Spectrum model estimates in July 2017 (*6*). The Spectrum model, which is updated each year, calculates annual estimates of the HIV epidemic, including HIV prevalence and ART coverage, to monitor changes in national epidemics. UNAIDS data were analyzed by year during 2012–2016, comparing national estimates of number of children aged <15 years with HIV infection with number of those children on ART for each country. All Spectrum country data were finalized in the 2016 model, with the exception of Lesotho and Zimbabwe, for which 2015 Spectrum estimates were used for the 2016 time point because 2016 UNAIDS estimates were not available.

The age at eligibility for ART among the 20 countries increased during 2012–2016; in 2012, 95% of countries included children aged <2 years. By 2016, 35% of countries included children aged <5 years and 65% included all children aged <15 years (Table). The 2013 WHO guidelines recommending ART eligibility for all children aged <5 years with HIV infection were adopted by six (30%) of the 20 countries in 2013; by 2014, 16 (80%) countries had adopted the guidelines, and by 2015, all 20 countries had implemented the policy. By the end of 2016, 13 (65%) of the 20 countries had adopted the 2016 WHO guidelines for ART eligibility for all children aged <15 years.

**TABLE:** Number and percentage of countries with age-specific pediatric ART eligibility, by year — 20 PEPFAR-supported sub-Saharan African countries,*^,†^ 2012─2016

Age at ART eligibility (year recommended by WHO)	Year				
	2012	2013	2014	2015	2016
	No. (%)	No. (%)	No. (%)	No. (%)	No. (%)
<1 yr	6 (30)	3 (15)	—^§^	—^§^	—^§^
<2 yrs (2010)	13 (65)	11 (55)	4 (20)	—^§^	—^§^
<5 yrs (2013)	1 (5)	6 (30)	12 (60)	15 (75)	7 (35)
<15 yrs (2016)	—^§^	—^§^	4 (20)	5 (25)	13 (65)

**Abbreviations:** AIDS = acquired immunodeficiency syndrome; ART = antiretroviral therapy; PEPFAR = U.S. President’s Emergency Plan for AIDS Relief; WHO = World Health Organization.

* Angola, Botswana, Cameroon, Côte d’Ivoire, Democratic Republic of the Congo, Ethiopia, Kenya, Lesotho, Malawi, Mozambique, Namibia, Nigeria, Rwanda, South Africa, South Sudan, Swaziland, Tanzania, Uganda, Zambia, and Zimbabwe.

**^†^** Kenya and Côte d’Ivoire adopted ART eligibility for children aged <10 years in 2014–2015 and 2015–2016, respectively; they were included in the <5 years category because they did not meet the more inclusive <15 years category criteria.

^§^ No country defined ART eligibility in that age range during that year.

The percentage of children with HIV infection receiving ART increased from 24% in 2012 to 44% in 2016 (Figure 1). However, although the ART coverage gap decreased steadily during 2012–2016, there were still approximately 750,000 (56%) children with HIV infection not on ART as of 2016.

**FIGURE 1:**
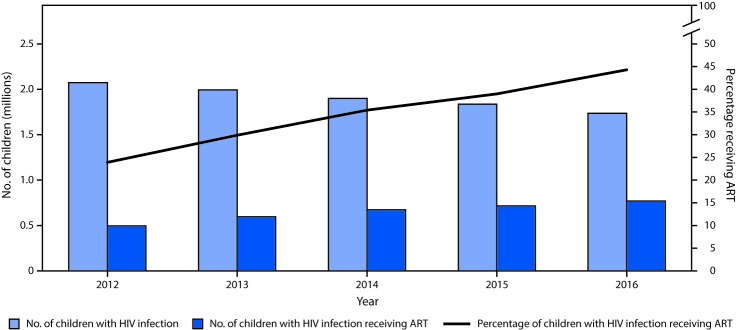
UNAIDS estimates for numbers of children with HIV infection and number and percentage receiving ART, by year — 20 PEPFAR-supported sub-Saharan African countries,*^,†^ 2012─2016 **Abbreviations:** AIDS = acquired immunodeficiency syndrome; ART = antiretroviral therapy; HIV = human immunodeficiency virus; PEPFAR = U.S. President’s Emergency Plan for AIDS Relief; UNAIDS = Joint United Nations Programme on HIV and AIDS. * Angola, Botswana, Cameroon, Côte d’Ivoire, Democratic Republic of the Congo, Ethiopia, Kenya, Lesotho, Malawi, Mozambique, Namibia, Nigeria, Rwanda, South Africa, South Sudan, Swaziland, Tanzania, Uganda, Zambia, and Zimbabwe. ^†^ Because 2016 UNAIDS estimates were not available for Lesotho and Zimbabwe, 2015 UNAIDS estimates were used for these two countries for 2016 time point.

In 2016, national pediatric ART coverage ranged from 5% in South Sudan to 66% in Namibia; coverage estimates for 11 of the 18 countries with available data were <50% (Figure 2). During 2012–2016, ART coverage among children with HIV infection increased in all countries, with the exception of Namibia. In four countries (Angola, Botswana, Rwanda, and South Africa), pediatric ART coverage increased by 50% or less, in seven (Cameroon, Côte d’Ivoire, Ethiopia, Kenya, Nigeria, Swaziland, and Zambia), coverage increased by 51%–100%, and in six (Democratic Republic of the Congo, Malawi, Mozambique, South Sudan, Tanzania, and Uganda), coverage increased by >100%.

**FIGURE 2:**
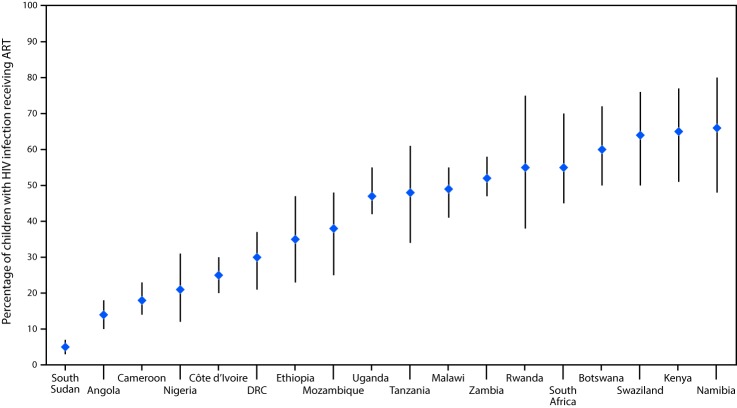
UNAIDS estimates of pediatric ART coverage — 18 PEPFAR-supported sub-Saharan African countries, 2016*^,†^ **Abbreviations:** AIDS = acquired immunodeficiency syndrome; ART = antiretroviral therapy; DRC = Democratic Republic of the Congo; HIV = human immunodeficiency virus; PEPFAR = U.S. President’s Emergency Plan for AIDS Relief; UNAIDS = Joint United Nations Programme on HIV and AIDS. * Angola, Botswana, Cameroon, Côte d’Ivoire, DRC, Ethiopia, Kenya, Malawi, Mozambique, Namibia, Nigeria, Rwanda, South Africa, South Sudan, Swaziland, Tanzania, Uganda, and Zambia. Lesotho and Zimbabwe were not included in the 2016 individual country analysis because 2016 UNAIDS estimates were not available. ^†^ The bars represent the ranges around the UNAIDS estimates and define the boundaries within which the actual numbers lie, according to UNAIDS.

## Discussion

As of 2015, all 20 PEPFAR-supported sub-Saharan African countries included in this analysis had adopted the 2013 WHO guidelines for ART eligibility for children with HIV infection aged <5 years. However, adoption of the 2013 guidelines in some countries did not occur until 2 years later, in 2015. Thirteen of the 20 countries expanded treatment eligibility at the end of 2016 to include all children aged <15 years, thus aligning with the most recent WHO guidelines published in 2016, which recommend a “treat all” approach.

Although the number of children on ART within these countries has risen steadily, a large coverage gap still exists between children with HIV infection and those who are receiving ART. Despite expanded ART eligibility guidelines, approximately 56% of children aged <15 years with HIV infection in these 20 PEPFAR-supported countries were not receiving life-saving ART in 2016. By country, the proportion of children with HIV infection on ART ranges from 5% to 66%.

Adoption of new guidelines requires review and approval by national experts and leaders, which often results in a lag between publication of WHO guidelines and country implementation of policy. Most low- and middle-income countries took almost 2 years to adopt the 2010 WHO ART guidelines (*7*). Limited data on the impact of early ART initiation in older age groups might also delay adoption of new guidelines for pediatric ART eligibility (*5*). Whereas ample data show reduced mortality with early ART for children aged <1 year, evidence for reduced mortality with early ART for children aged ≥1 year are limited; however, benefits of early ART initiation in older children for growth, neurodevelopment, and retention in care have been identified (*5*,*8*).

Pediatric ART uptake challenges also include variable funding and procurement of pediatric ART formulations, ongoing need for training of clinical staff on current guidelines, and pediatric ART acceptance and administration by caregivers (*7*,*9*). Adoption of the most effective pediatric ART regimens and formulations requires additional agreement among national experts, guideline formulation, and implementation.

Although prompt adoption and implementation of expanded ART eligibility is required for improved pediatric ART coverage, attention to other key components of the HIV treatment cascade is critical. Identification of all children with HIV infection is fundamental; therefore, active case finding is essential to identify undiagnosed children with HIV infection included in the UNAIDS estimates. Prompt linkage to care (ideally, with availability and initiation of same-day ART) is also required to initiate ART for patients with newly diagnosed HIV infection. Lastly, community-based retention and adherence support are necessary to help maintain children with HIV infection on ART; patient tracking might help initiate ART for previously ineligible children or those lost to follow-up (*9*).

The findings in this report are subject to at least two limitations. First, national guidelines were assessed based on the publication date, which might not match the date of policy implementation. Second, UNAIDS estimates for children with HIV infection and those on ART are derived from complex models. The most recent model estimates were used, but refinement occurs yearly, and estimates will continue to change. The Population-based HIV Impact Assessments, a collaboration between PEPFAR and ministries of health, are providing more accurate measures of HIV prevalence and incidence to incorporate into future UNAIDS models (*10*). Because 2016 UNAIDS estimates were not available for Zimbabwe and Lesotho, 2015 estimates were used, which likely slightly overestimated the number of children with HIV infection and underestimated the number on ART and ART coverage.

This report highlights the continuing gaps in pediatric ART coverage in PEPFAR-supported sub-Saharan African countries with high HIV burden, despite expanded ART eligibility criteria. Robust pediatric HIV testing and comprehensive ART programs are needed to ensure that all children with HIV infection are identified and initiated on ART as early as possible. Further evaluation might identify challenges to implementation of ART guidelines and help rapidly address this gap in pediatric ART coverage to further reduce morbidity and mortality among children with HIV infection.

**Summary**

**What is already known about this topic?**

World Health Organization (WHO) guidelines have expanded the recommended criteria for life-saving antiretroviral therapy (ART) eligibility among children with human immunodeficiency virus (HIV) infection.

**What is added by this report?**

All 20 sub-Saharan African countries included in this analysis adopted the 2013 WHO guidelines by 2015. In 2016, 13 of 20 countries adopted the 2016 guidelines to treat all children; however, approximately 56% of children aged <15 years with HIV infection in these countries were not receiving ART.

**What are the implications for public health practice?**

Closing the ART coverage gap requires prompt adoption of WHO guidelines, and strengthening ART programs to identify children with HIV infection, link them to HIV treatment programs, and ensure their retention in care.

## Footnotes

* Angola, Botswana, Cameroon, Côte d’Ivoire, Democratic Republic of the Congo, Ethiopia, Kenya, Lesotho, Malawi, Mozambique, Namibia, Nigeria, Rwanda, South Africa, South Sudan, Swaziland, Tanzania, Uganda, Zambia, and Zimbabwe.
